# Neuroanatomy of the nodosaurid *Struthiosaurus austriacus* (Dinosauria: Thyreophora) supports potential ecological differentiations within Ankylosauria

**DOI:** 10.1038/s41598-021-03599-9

**Published:** 2022-01-07

**Authors:** Marco Schade, Sebastian Stumpf, Jürgen Kriwet, Christoph Kettler, Cathrin Pfaff

**Affiliations:** 1 Institute of Geography and Geology, Palaeontology and Historical Geology, University of Greifswald, 17489 Greifswald, Germany; 2 Zoological Institute and Museum, Cytology and Evolutionary Biology, University of Greifswald, 17489 Greifswald, Germany; 3 Department of Earth and Environmental Sciences, Palaeontology and Geobiology, Ludwig-Maximilians-Universität, 80333 Munich, Germany; 4 Department of Palaeontology, Faculty of Earth Sciences, Geography and Astronomy, University of Vienna, 1090 Vienna, Austria; 5 Department of Geology, Faculty of Earth Sciences, Geography and Astronomy, University of Vienna, 1090 Vienna, Austria

**Keywords:** Palaeontology, Animal behaviour, Behavioural ecology

## Abstract

Nodosauridae is a group of thyreophoran dinosaurs characterized by a collar of prominent osteoderms. In comparison to its sister group, the often club-tailed ankylosaurids, a different lifestyle of nodosaurids could be assumed based on their neuroanatomy and weaponry, e.g., regarding applied defensive strategies. The holotype of the nodosaurid *Struthiosaurus austriacus* consists of a single partial braincase from the Late Cretaceous of Austria. Since neuroanatomy is considered to be associated with ecological tendencies, we created digital models of the braincase based on micro-CT data. The cranial endocast of *S. austriacus* generally resembles those of its relatives. A network of vascular canals surrounding the brain cavity further supports special thermoregulatory adaptations within Ankylosauria. The horizontal orientation of the lateral semicircular canal independently confirms previous appraisals of head posture for *S. austriacus* and, hence, strengthens the usage of the LSC as proxy for habitual head posture in fossil tetrapods. The short anterior and angular lateral semicircular canals, combined with the relatively shortest dinosaurian cochlear duct known so far and the lack of a floccular recess suggest a rather inert lifestyle without the necessity of sophisticated senses for equilibrium and hearing in *S. austriacus*. These observations agree with an animal that adapted to a comparatively inactive lifestyle with limited social interactions.

## Introduction

Thyreophora are ornithischian dinosaurs, comprising iconic taxa like *Stegosaurus* and *Ankylosaurus*^1^. Ankylosauria thrived at least since the Middle Jurassic and some of their representatives witnessed the end-Cretaceous mass extinction^1^. These globally distributed quadruped herbivores were heavily armoured living fortresses; partly either equipped with a club tail (ankylosaurids^2^) or a collar of hypertrophied spikes on their neck and shoulders (nodosaurids^3^). Potential palaeoenvironmental^4^ and food preferences^5^, together with features of their nasal passages^6^, jaw mechanics^7,8^ and osteoderms^9^, may indicate different lifestyles for both groups.

Since the brain and associated neuroanatomical structures of vertebrates leave perceivable traces, which are possibly ecologically informative, it is worthwhile to thoroughly examine the braincase of nodosaurids in order to compare it to ankylosaurids. Whereas complete braincase material among early-diverging thyreophorans is only known from *Scelidosaurus harrisoni*^10^, neurocranial material of stegosaurs^11,12^, and ankylosaurs (e.g.^13–15^) is more common. The heavily armored skull roofs of the latter likely improved their preservation potential.

*Struthiosaurus* is a European nodosaurid with an estimated body length of up to three metres, known from cranial and postcranial material of Campanian to Maastrichtian age^16–22^. As currently accepted, *Struthiosaurus* comprises three species: *S*. *austriacus* from the early Campanian of Austria^16–18^, *S*. *languedocensis* from the early Campanian of France^22^, and *S*. *transylvanicus* from the Maastrichtian of Romania^23,24^. In addition, skeletal remains referred to *Struthiosaurus* sp. were reported from late Campanian to early Maastrichtian deposits of Spain^20,21^. The potentially oldest fossil record of *Struthiosaurus* is represented by a single right humerus from the Santonian of Hungary^25^. The type species of *Struthiosaurus*, *S*. *austriacus*, is based on fragmentary cranial and postcranial remains of at least three individuals of different ontogenetic stages that were recovered during the nineteenth century from early Campanian continental coal-bearing beds of Muthmannsdorf, Austria, referred to the Grünbach Formation (see^18^ for overview).

**Figure 1:**
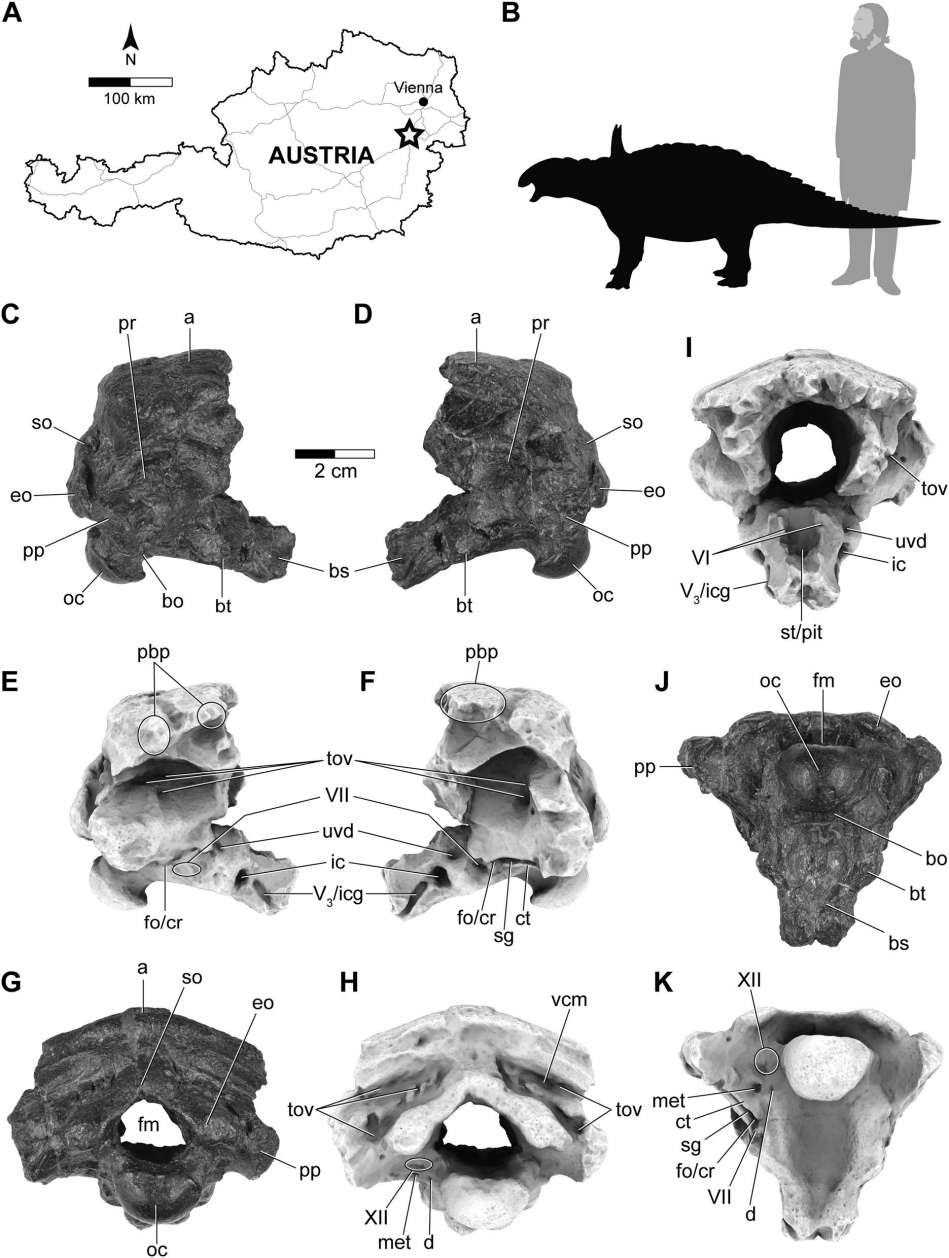
(**A**) Outline drawing of Austria with a star marking Muthmannsdorf, the type locality of *Struthiosaurus austriacus*. (**B**) Silhouette of *Struthiosaurus austriacus* (measuring 2.7 m in length here; copyright: Fabrizio De Rossi) and a human for comparison. Photographs (**C**,**D**,**G**,**J**) and ambient occlusion photogrammetric models (**E**,**F**,**H**,**I**,**K**) of the holotype specimen of *Struthiosaurus austriacus*, IPUW 2349/6, in (**C**,**E**) right lateral, (**D**,**F**) left lateral, (**I**) anterior, (**J**,**K**) ventral and (**G**,**H**) posterior views. a, armour; bo, basioccipital; bs, basisphenoid; bt, basal tuber; ct, crista tuberalis; d, damage; fm, foramen magnum; fo/cr, fenestra ovalis/columellar recess; met, metotic foramen; pbp, posterior branching plexus; sg, stapedial groove; st/pit, sella turcica/pituitary; tov, transverso-occipital vein; uvd, uncertain vascular duct; V_3_/icg, groove for the mandibular branch of the trigeminal nerve or for the internal carotid; VI, abducens nerve; VII, facial nerve; vcm, dorsal middle cerebral vein; XII, hypoglossal nerve.

The holotype specimen of *S. austriacus*, a partial braincase (IPUW 2349/6; Fig. 1), was scanned with the aid of a micro-CT; its superficial morphology has been previously described^16,17,26^. The segmentation of the internal structures provides new insights into the neuroanatomy and behavioral capacities of this Late Cretaceous (Campanian) armoured dinosaur from Austria.

## Results

### Cranial endocast, innervation and blood supply

**Figure 2:**
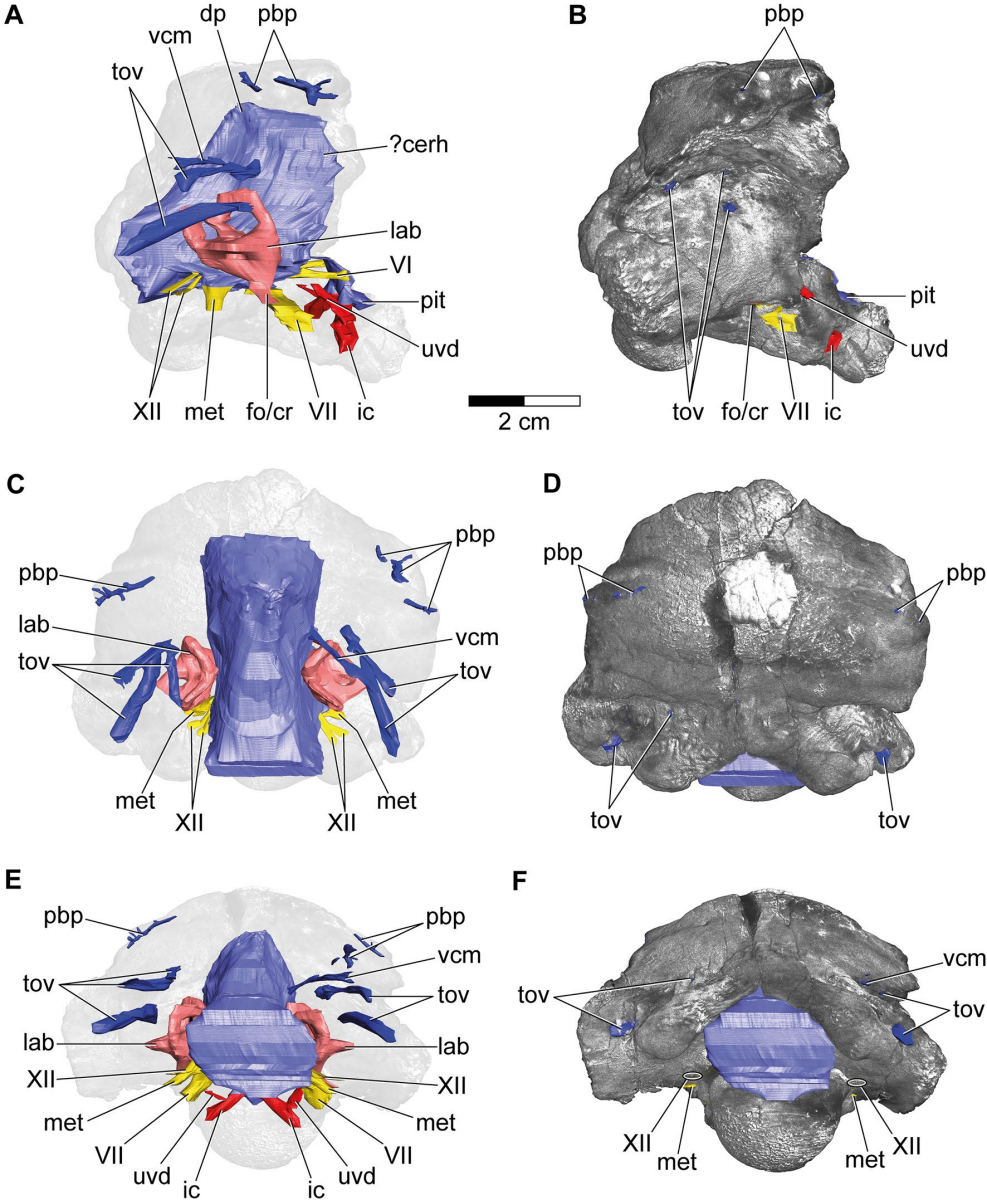
3D model of the cranial endocast with endosseous labyrinths and neurovascular canals of the holotype specimen of *Struthiosaurus austriacus*, IPUW 2349/6, without (**A**,**C**,**E**) and with (**B**,**D**,**F**) a volume rendering of the braincase in (**A**,**B**) right lateral, (**C**,**D**) dorsal and (**E**,**F**) posterior aspects. ?cerh, possible cerebral hemisphere; dp, dural peak; fo/cr, fenestra ovalis/columellar recess; met, metotic foramen; lab, endosseous labyrinth; pbp, posterior branching plexus; pit, pituitary; tov, transverso-occipital vein; uvd, uncertain vascular duct; VI, abducens nerve; VII, facial nerve; vcm, dorsal middle cerebral vein; XII, hypoglossal nerve.

**Figure 3:**
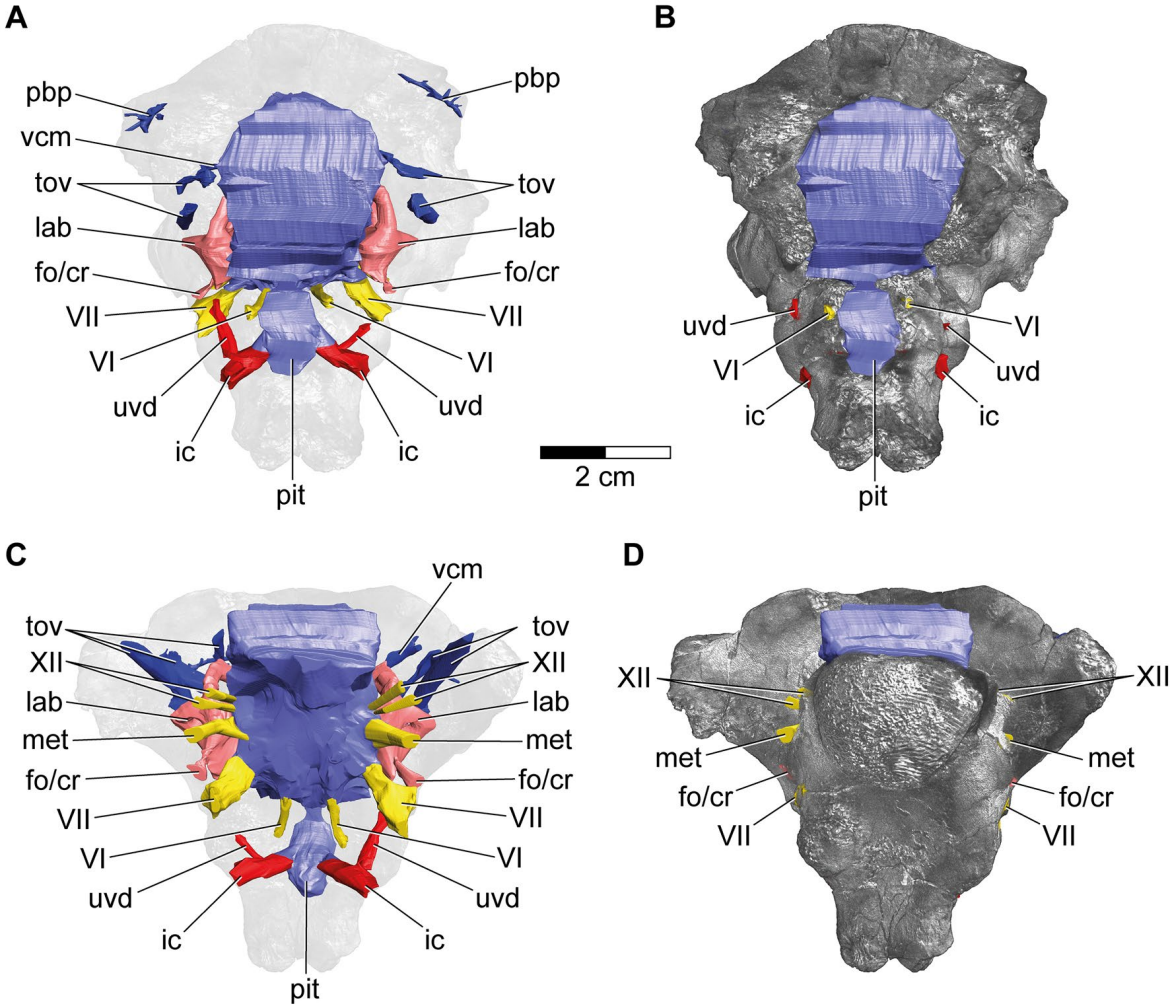
3D model of the cranial endocast with endosseous labyrinths and neurovascular canals of the holotype specimen of *Struthiosaurus austriacus*, IPUW 2349/6 without (**A**,**C**) and with (**B**,**D**) a volume rendering of the braincase in (**A**,**B**) anterior and (**C**,**D**) ventral views. fo/cr, fenestra ovalis/columellar recess; met, metotic foramen; lab, endosseous labyrinth; pbp, posterior branching plexus; pit, pituitary; tov, transverso-occipital vein; uvd, uncertain vascular duct; VI, abducens nerve; VII, facial nerve; vcm, dorsal middle cerebral vein; XII, hypoglossal nerve.

As in most non-maniraptoriform dinosaur braincases (e.g.^27–29^), features of the midbrain and hindbrain are not securely identifiable as imprints on the endocast of IPUW 2349/6 (Figs. 2, 3). This suggests little correlation of the brain and respective soft tissues with the surface of the endocranial cavity in the living animal, which is similar to extant reptiles (e.g., crocodiles and turtles^30,31^).

Although not completely preserved, the endocranial cavity suggests great angles of both the cerebral and the pontine flexure in IPUW 2349/6, matching the condition in other ankylosaurs and stegosaurs^11–13,32^. The endocast shows a slight dural peak (Fig. 2A), where the pineal gland would be expected, similar to the condition in the ankylosaur *Kunbarrasaurus ieversi*^14^. The presence of the cartilage-filled pit in the supraoccipital, expressed as an anteroventrally inclined ridge on the cranial endocasts of *Struthiosaurus austriacus*, *S*. *transylvanicus*^17^ and the nodosaurid *Hungarosaurus* sp.^32^, is barely discernable in the digital endocast of IPUW 2349/6. Ventral to the pineal, around the mid-height of the brain endocast, the dorsal middle cerebral vein could be reconstructed on the right side (Fig. 2E,F; vcm). Additionally, at least two large canals, tentatively assigned to the transverso-occipital vein^13^, traverse each paroccipital process and the prootic mainly anteroposteriorly; without any obvious connection to another endocranial structure (Fig. 2A,C,E). More delicate and complex networks of canals are present further anterodorsally and may belong to the posterior branching plexus^13^. Possibly, the expanding mediolateral width of the anterodorsal endocast marks the otherwise barely delineated cerebral hemispheres. Anteroventrally, the posterior portion of the pituitary fossa is preserved, together with the ventrolaterally directed internal carotid artery (Fig. 3A,B; pit; ic). Around the preserved mid-length, each internal carotid artery branch is connected to another vascular duct of uncertain identity, leading in a posterodorsolateral direction. The canal for the abducens nerve (Fig. 3A,B; CN VI) is situated posterolaterally to the pituitary fossa. While the facial nerve opening (CN VII) is situated anteriorly to the cochlear duct, the columellar recess, the metotic foramen (for CN IX-XI and jugular vein) and the hypoglossal nerve openings (CN XII) are situated posterior to the cochlear duct. Due to the fact that the specimen is strongly traversed by breakages, the canals for CN VII and IX-XI likely appear slightly too large in our reconstruction, but certainly approximate the course of the respective canals. However, for the same reason, the exact position and course of the CN XII openings and canals could not be established with certainty, but are estimated on the basis of internal and external characteristics. The micro-CT data suggest the presence of two CN XII openings per side, which are largely obscured by cracks on the outside (Figs. 1J,K, 2E,F, 3C,D). While Pereda-Suberbiola and Galton^16^ identified two foramina on the left side of the specimen as the openings for CN XII, our reconstruction suggests that the more ventrally situated foramen actually represents the metotic foramen. In posterior view, medially to the left metotic foramen, another foramen seems to be situated (Fig. 1J,K) but the micro-CT data show that this is just a superficial damage. The midbrain of IPUW 2349/6 does not possess a floccular recess. Posteriorly, the foramen magnum is connected to the mediolaterally wide medulla oblongata. The incomplete endocranial cavity comprises a volume of around 12 cm^3^ (measured according to Sampson and Witmer^33^).

### Endosseous labyrinth

**Figure 4:**
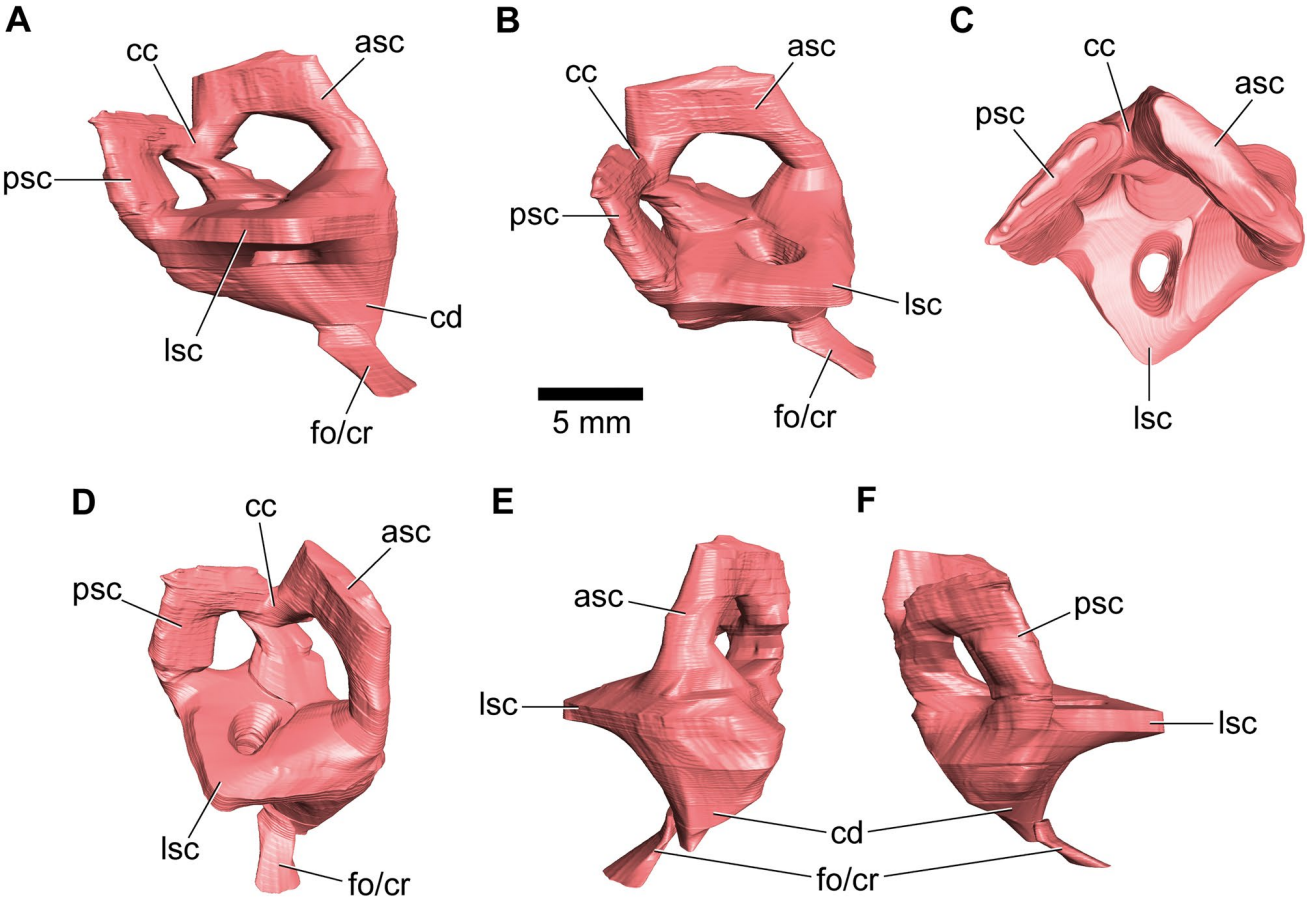
3D model of the right endosseous labyrinth of the holotype specimen of *Struthiosaurus austriacus*, IPUW 2349/6, in (**A**) lateral, (**B**) posterodorsolateral, (**C**) dorsal, (**D**) anterodorsolateral, (**E**) anterior and (**F**) posterior views. asc, anterior semicircular canal; cc, common crus; cd, cochlear duct; fo/cr, fenestra ovalis/columellar recess; lsc, lateral semicircular canal; psc, posterior semicircular canal.

The vestibular system is ventrally connected to the cochlea and together they form the completely preserved endosseous labyrinth of IPUW 2349/6 (Fig. 4). The anterior semicircular canal is dorsoventrally slightly higher and anteroposteriorly wider than the posterior semicircular canal (Fig. 4A). The vertical semicircular canals are thick and short in relation to the complete vestibular system. In dorsal view, they enclose an angle of 91° in the right vestibular system and 98° at the left vestibular system. The common crus, uniting the anterior and posterior semicircular canal, is slightly posteriorly inclined. In dorsal view, the relatively thick lateral semicircular canal projects posterolaterally and arcs towards the posterior ampulla, producing a very acute angle on its distal-most corner (Fig. 4C). The cochlear duct is strikingly short on both sides, being dorsoventrally shorter than the vestibular system (Fig. 4E). The cochlear duct projects anteroventrolaterally and is strongly tapered distally.

### Auditory capabilities

In order to very roughly estimate the auditory capability of *S. austriacus*, we digitally measured the dorsoventral cochlear duct length (c. 5.9 mm; as outlined by Walsh et al.^34^) and the anteroposterior basicranial length (c. 40.5 mm; measured between the anterior-most preserved part of the sella turcica and the distal-most part of the occipital condyle). Following the equations of Walsh et al.^34^, we calculate the mean hearing frequency of *S. austriacus* as 1230 Hz and the frequency bandwidth as 1868 Hz (between 296 and 2164 Hz).

## Discussion

### Neurovascularanatomy and ecological affinities

The presence of widely distributed vascular canals in the holotypic neurocranium of *Struthiosaurus austriacus* adds to the diversity of patterns within Thyreophora^6,13,14,35^. Conversely to *Bissektipelta archibaldi*^13^, these canals are not obviously interconnected with each other or the cavity that once contained the brain, and are located closer to the endosseous labyrinth in *S. austriacus.* As other authors suggested^6,13,35^, a complex network of blood supply tissue within the braincase could have contributed to remodelling of skull bones and armour, and thermoregulation of the brain. Additionally, the posterodorsolaterally directed duct on each internal carotid artery branch found in *S. austriacus* likely played a role in thermoregulation^35^.

The endosseous labyrinth of vertebrates detects head movements, making it critical in gaze stabilization during locomotion (e.g.^36^). Currently, it is a matter of debate to what extent the morphology of the semicircular canals is a proxy for certain ecological affinities within Archosauromorpha^37,38^. Few endosseous labyrinths of ankylosaur taxa are known, of which all possess relatively short and thick semicircular canals, as seen in the early-diverging ankylosaur *Kunbarrasaurus ieversi*^14^, the ankylosaurids *B. archibaldi*^13^, *Euoplocephalus tutus*^39^ (and probably *T. teresae*^15^), and the nodosaurid *Pawpawsaurus campbelli*^40^. Hence, including *S. austriacus*, only two nodosaurid endosseous labyrinths are known to date and both display an anterior semicircular canal, which is just slightly longer than the respective posterior semicircular canal. This contrasts the condition seen in *K. ieversi*^14^, *B. archibaldi*^13^ and *E. tutus*^39^, clearly showing a relatively longer ASC. Long semicircular canals are thought to be more sensitive for head movements^33^, and hence potentially related to neck mobility^41,42^. The unique and conspicuously acute angle at mid-length of the lateral semicircular canal of *S. austriacus* did probably impede a continuous endolymphatic flow, causing insensitivity in comparison to the usual rounded condition. Furthermore, the combination of a longer ASC in two ankylosaurid taxa (*B. archibaldi* and *E. tutus*), as well as the presence of a floccular recess (*E. tutus* and *T. teresae*), may render ankylosaurids superior in VOR (vestibulo-ocular reflex) and VCR (vestibulo-collic reflex) procession in comparison to nodosaurids. This could be associated with a more active kind of protective behaviour in ankylosaurids, involving digging and targeting usage of their tail clubs^2,15,43,44^.

Because of its involvement in processing VOR/VCR, the flocculus is a critical structure of the cerebellum for control and coordination of head, eye, and neck movements^41,45,46^. Although the size of the floccular fossa has been found to fail as a proxy for ecology or behavior in certain extant mammals and birds^47^, it has repeatedly been used to tentatively establish such a meaning for fossil taxa (e.g.^15,33,48^). Additionally, ontogeny possibly plays a role in the expression of the flocculus on the endocast^28^. A lack of a floccular recess is common for ankylosaurs, except for a group within Ankylosaurinae^13,15^, and no floccular recess has been found in any nodosaurid endocast so far^32,40^. However, a floccular recess is present in the ankylosaurine ankylosaurids *E. tutus*^14,39^ and *T. teresae*^40^. Furthermore, braincase endocasts of the stegosauruids *Stegosaurus*^11^ and *Kentrosaurus*^12^ share slight lateral eminences, which have been identified as floccular recesses (however, not present in all *Stegosaurus* specimens^14^), and both taxa have spiked tails, which were very likely proper defensive means^49,50^. Arbour and Currie^2^ reported a stepwise acquisition of clubbed tails in ankylosaurids, leading to a handle first (interlocking vertebrae produce a stiffened tail) and a knob second (fusion of distal-most osteoderms) model. Just like nodosaurids, some early-diverging ankylosaurids show no handle or knob adaptations^2^. Only ankylosaurine ankylosaurids show both, a handle and a knob, producing a functional tail club^2^. It is conspicuous that endocasts of thyreophoran taxa with a formidable weapon on the tail (*Stegosaurus*, *Kentrosaurus*, *Euoplocephalus* and *Tarchia*; although a tail club has only been referred to *Tarchia*^2,51^) bear a floccular recess as well^11,12,39,40^. In contrast, nodosaurids and early-diverging ankylosaurids neither show a distinct floccular recess^13,32,40^, nor a tail club^2^ for which targeting would have been useful. Nodosaurids bear long spikes around their neck and shoulder^3^ (which likely rather limited their neck mobility^42^) in addition to osteoderms with relatively thicker cancellous cores^52^; an involvement in thermoregulation and display has been hypothesized for ankylosaur armor^9^. While ankylosaurids tend to bear armours only with bulky osteoderms, nodosaurids additionally possess proximodistally very elongated elements^3,53^, may producing an armour with a comparably passive protective/offensive utility^9^.This may suggest a more passive defense tactic through simple hunker down behavior in nodosaurids with less reliance on coordination-related (VOR/VCR) neural tissue (in contrast to^32^). Regarding the demonstrably well vascularized neurocranium of ankylosaurs^6,13^, the putative hypertrophied cerebellum in endocasts of *Hungarosaurus* sp.^32^ and *Struthiosaurus transylvanicus*^17^ may rather represent areas of extensive blood supply and other soft tissues. Possibly, the flocculus independently developed a larger size because of its neurologic involvement^54^ in VOR/VCR in the actively defending stegosaurs and late-diverging ankylosaurids (ankylosaurines).

The orientation of the lateral semicircular canal as a proxy for head posture is not necessarily straightforward^55,56^. However, our reconstructions suggest a strongly posteroventrally inclined occipital condyle (c. 55°; Figs. 1C, 2B) for *S. austriacus* when the LSC is horizontally arranged. Hence, this independently supports the findings of Pereda-Suberbiola and Galton^17^, who compared the skull roof and basisphenoid of *S. austriacus* with *Panoplosaurus mirus*, signifying a habitually slightly inclined snout in both taxa.

### Auditory capacities and sound production

Following the procedure of Walsh et al.^34^, the auditory acuity of *S. austriacus* seems somewhat superior to that of turtles. Paulina-Carabajal et al.^40^ considered the cochlea to be relatively short in the nodosaurid *P. campbelli* because of the ventrally situated fenestra ovalis, which marks the border of the vestibular system and the cochlea. However, this contrasts with the practice of Walsh et al.^34^, who included the fenestra ovalis in the cochlear duct length. Animals are likely to perceive sounds they are able to produce themselves (Walsh et al.^34^ and references therein). Because ankylosaurids seem to possess longer cochlear ducts than nodosaurids, it has been hypothesized that ankylosaurids had more sophisticated sound producing and perception capabilities in comparison to nodosaurids^15,40^. The presence of relatively shorter and less convoluted nasal passages, which are possibly involved in sound production, in the nodosaurid *P. mirus*, compared to the ankylosaurid *E. tutus*, potentially supports this interpretation^6,30,40,42^. Nonetheless, the nasal passages of ankylosaurs may have mainly served as adaptation for thermal homeostasis of the brain by vascular tissues shedding excess heat into the nasal passages (being seemingly more efficient in *E. tutus* than in *P. mirus*^6^). The extremely short cochlear duct of *S. austriacus* (in fact the shortest found in a dinosaur so far) may further support inferior auditory capabilities of nodosaurids in comparison to ankylosaurids.

## Conclusions

Whereas nodosaurids and ankylosaurids were lumbering^57,58^, heavily armoured^9,52^ and low-browsing^5^ animals, mainly relying on large guts (possibly for fermentation) to digest^53^, nodosaurids possibly preferred coastal or fluvial environments^4^ and are suspected for having evolved jaw biomechanics delivering stronger bite forces^7,8^ (potentially for tougher plant material), and the gut content of a nodosaurid hints to a selective feedings style^5^.Furthermore, the combination of a relatively short cochlear duct^40^, the lack of a floccular recess^13,17,32,40^, a short ASC^40^, less elaborated nasal passages^6^, the obligate absence of a tail club^2^, but thickened osteoderms^9,52^ in nodosaurids indicate different ecological adaptations in comparison to ankylosaurids. Hence, nodosaurids were possibly less reliant on their sense of hearing, applied a less active style of self-defense and, apparently, occupied different ecological niches than ankylosaurids. The new findings of the neuroanatomy of *Struthiosaurus austriacus* seem to add to this differentiation.

## Materials and methods

The holotype specimen of *Struthiosaurus austriacus*, IPUW 2349/6, represents an incomplete braincase that is traversed by breakages but not deformed, preserving the posterior part of the skull roof, most of the occipital region and part of the basicranium. It is about 55 mm in mediolateral width and measures 50 mm anteroposteriorly and dorsoventrally. Although already described elsewhere^16,17,26^, micro-CT-based neuroanatomical accounts for IPUW 2349/6 have never been made.

We scanned IPUW 2349/6 using the desktop micro-computed tomography device (micro-CT) SkyScan/Bruker 1173 housed in the Department of Palaeontology, University of Vienna (voltage: 130 kV, X-ray tube current: 61 μA, exposure time: 1249 ms, filter: brass 0.25 mm, voxel size: 0.032904 mm). Digital segmentation and measurements were produced utilizing the software Amira (6.1), based on bmp image files, which were exported using DataViewer 1.5.4.0. (Skyscan/Bruker). The micro-CT data were manually segmented to create 3D models, which were mirrored afterwards. The density contrast between the fossil and the sediment within was relatively weak, but whereas the respective cavities were still clearly discernable, it was not possible to distinguish individual bones and sutures of the neurocranium. The photogrammetry models are based on 122 photographs and were created with Agisoft (1.7.2).

## Data Availability

The micro-CT slice data, neuroanatomical and photogrammetry models of IPUW 2349/6, are published online, in the repository MorphoSource (Project: Struthiosaurus austriacus-IPUW 2349/6-Schade et al. 2021 neuroanatomy//MorphoSource).
